# Clinical Cases

**Published:** 1887-04

**Authors:** J. T. Kent

**Affiliations:** St. Louis, Mo.


### CLINICAL CASES.

PROFESSOR J. T. KENT, M. D., ST. LOUIS, MO.

### ULCER ON THE LEG.—PULSATILLA.

Mrs. W., age seventy-three, writes: "The first breaking out of the ulcer she felt a smarting and stinging pain in her left ankle; there was a little elevation the size of a pea; the next day it broke and discharged a thin, bloody pus; around it was a purplish red color. The sore kept extending, also the discolored surface; then came a thick, yellow discharge of pus. The ulcer is now somewhat larger than a silver dollar. The surface of the ulcer looks like a sponge, and very red, covered with yellow, lumpy matter; the outside is almost on a level with the sore, I should say flat. The cloth that comes off (with mutton tallow) is slightly offensive; the ulcer I can scarcely smell; it burns, stings, and smarts; sometimes has a jerking sensation through the heel. She pulls her skirts up to cool the limb, which is better in the cool air. The warmer it is the worse it smarts and burns. Sometimes she describes the pain as something like splinters. From the knee down the leg sweats so that the hose is constantly wet. The well one is not so. As she gets up in the morning the foot swells until it is full and pains her very much; about three or four P. M. she gets easier and can lie down with some comfort. When she elevates the foot it feels much better, and does not swell so, and she is quite free from pain."

She has also some rheumatic symptoms that I suppose you want to know. There is great soreness from the shoulder to the elbow, and also in the cords of the neck. If she fans herself or uses her arms she has great pains in these parts. The upper arm aches with a grumbling, burning pain; she cannot put her arms back; both sides are alike. She can hold her hands over her head, but cannot reach out for anything. The fingers are swelled and stiff in the morning; the left hand is worse than the right. She often holds on to one arm, then the other; when she turns in bed she has to fold the arms and then work herself over. She is thirsty and feverish in the *afternoon.*

Puls.$^{cm}$ (H. S.), one dose, was immediately mailed to the patient, who lives nearly three hundred miles from this city.

Several watery stools followed, and all her symptoms were made worse, but she has many times taken a homœopathic remedy, and she remarked to her daughter that she was now going to recover again.

This leg ulcer is an old relic of barbarism with her, as she had had it cured several times allopathically. Some years ago I healed it with Sulph. very high, but it had to come again. The ulcer and the concomitants all departed in due time, and she is a picture of health now. The ulcer has been healed a year now, and she has not taken a dose of medicine since the Puls. mentioned. I am informed that at the end of six weeks the ulcer was healed. The potency was made by Dr. Johnstone on his new potentizer, *Hahnemann scale;* none are more reliable. The power to cure is in every one of these potencies. Every follower of Hahnemann should use the word "potency," and discard the words "attenuation" and "dilution."

When compelled to prescribe on a letter written by a laywoman, many things are wanting, but in the above we have the picture as given—no more and no less. The remedy was sent, and the patient, after all her family had settled down to this as her last sickness, made a good recovery. This is not the exception, but the rule after such prescriptions. If experience is appealed to or theory or *cures*, the inductive method must give us safest practice.

### SICK HEADCHE.—LAC-DEF., LAC-CAN.

1886, July 10th.—Mrs. R. S., widow, aged thirty-five. "I have had sick headaches many years." Had peritonitis, had typhoid fever, and was down in bed four months. These headaches have been coming ever since, now five years.

Headache back of eyes.

Sunlight brings on the headache.

"If I go without eating I have a headache."

"If I eat too much I have a headache."

"Excitement brings on the headache."

"Going to theatre brings on the headache."

"I have lain three and four days in a dark room, not able to endure any light."

Milk brings on the sick headache.

Eating never relieves the headache.

"When sick with typhoid fever I was fed on milk until I vomited whenever they brought it to my bed."

"I am never free from headache, but I am able to be at my desk about one-half of my time, much of which I suffer intensely." Seldom vomits, but much nausea.

"When I vomit it is of the food eaten, sour and bitter."

Here are the symptoms. What is the remedy?

It was evident that I had no ordinary case on hand, as two good prescribers had failed to help and told her so. Many whom I do not regard as careful physicians had treated her also. If the remedy must out, here it is : Lac-def.$^{cm}$ (Fincke).

July 16th.—She returned. Just finished, one of the most violent headaches ever had. "So sleepy while writing my letters that could hardly hold my eyes open." Had to quit work two afternoons and go home. I am greatly discouraged when the headache is on. Sleepy while writing is new. Sac. lac.

July 23d.—No headache since last call.

July 30th.—A short headache, but feeling better. Lac-def.$^{cm}$ (F.), dry, one dose.

August 2d.—Headache came, lasted two days, but has heretofore generally lasted a week. Improving generally. Lac-def.$^{cm}$ (F.)

August 10th.—Improving.

August 26th.—Improving; has just finished a headache, but went three weeks.

September 11th.—Headache in two weeks. Symptoms about as usual. Sulph.$^{cm}$, one dose (Fincke).

September 12th.—Headache is on full force, started in left eye, sunlight makes it worse. "I felt the headache this time from delaying my dinner." Gnawing, hungry feeling, not relieved by eating. Everything I eat makes me worse but fish. Cold brings on headache. Headache worse from weight of hat. This headache began in left eye and has extended to occiput. "The thought of milk makes me sick." No palliative was given, but watching the symptoms seemed to be the only way of finding the remedy.

September 13th.—Reports that the afternoon of yesterday, the 12th, headache went over to right eye and side of head, but now it is back in my left eye. Lac-can.$^{mm}$ (Swan) was given, and immediately relief followed, and it was three months before another came, and it was very short and did not compel her to leave her desk.

February 10th.—She had a slight headache and took another dose of Lac-can.$^{cm}$ (F. C.) She has been compelled to lay aside all her clothing and procure larger size. Can eat anything, and enjoys life like other people.

---

Visions on closing the eyes, Argent-nit., Bell., Bry., Calc.-c., China, Ig., Thuja.

This symptom is believed by many physicians to be peculiar to Bell. It will thus be seen that other remedies have it. The authority, if desired, can be given for each remedy.

